# Manipulation of Senescence of Plants to Improve Biotic Stress Resistance

**DOI:** 10.3390/life12101496

**Published:** 2022-09-26

**Authors:** Balázs Barna

**Affiliations:** Plant Protection Institute, Research Centre for Agriculture, ELKH, 1022 Budapest, Hungary; barna.balazs@atk.hu

**Keywords:** biotic stress tolerance, plant hormones, antioxidants, membrane lipids, in vitro selection, mutagenesis, transgenic plants, mycorrhiza

## Abstract

The physiological state, i.e., senescence or juvenility, of plants and plant organs can have strong effect on their reactions to pathogen attacks. This effect is mainly expressed as changes in the severity of disease symptoms. Generally, necrotrophic pathogens cause more severe symptoms on senescent than on juvenile plants, while biotrophs prefer juvenile tissues. Several factors of senescence have opposite effect on the two pathogen groups, such as decreased photosynthesis, decreased antioxidant capacity, remobilization of nutrients, changes in plant hormonal network, and in fluidity of cell membranes. Furthermore, senescent tissues are less tolerant to toxins and to cell-wall-degrading enzymes. On the other hand, pathogen infection itself has significant effect on the physiology of plants depending on the lifestyle of the pathogen and on the compatibility or incompatibility of the interaction with the plant. There are several possibilities to manipulate the physiological state of plants in order to improve their biotic and abiotic stress tolerance, such as removal of the terminal bud or high doses of nitrogen, external application of cytokinins or of inhibitors of ethylene action, as well as by spontaneous or directed mutation, in vitro selection, or manipulation by various transgenic approach. Even application of mycorrhiza can inhibit the senescence process of plants and improve their tolerance to stresses.

## 1. Introduction

It has been known for a long time that the physiological state, i.e., juvenility or senescence, of plant organs and tissues, either natural or induced by hormone treatment, can have strong influence on their susceptibility or resistance to pathogens [1,2,3,4,5,6,7]. Inhibition or promotion of senescence generally does not change qualitatively but rather quantitatively the relationship between the plant and the pathogen. Namely, a compatible interaction will not be incompatible or vice versa: a resistant leaf will not be fully susceptible, but the development of the pathogen and the severity of symptoms (number and size of pathogen colonies or lesions, etc.) could be increased or suppressed by changes of the leaf senescence process. It is important to note that senescence-associated resistance is different from the so-called *adult plant resistance* (APR). Adult plant resistance is the phenomenon in which disease-resistance genes are able to confer resistance at the adult stages of the plant, but they fail to do so at the seedling stages [8]. Another similar but different category is the *predisposition*. Predisposition is defined as treatments and conditions acting before inoculation to affect susceptibility to biotic and abiotic stresses. In other words, predisposition results from abiotic stresses occurring prior to infection that affect susceptibility of plants to disease [9].

The effect of the age of a plant organ or tissue on the plant–pathogen interactions strongly depends on the pathogen lifestyle. Generally, biotrophic pathogens, such as rusts, powdery mildews, or Peronospora, which get their nutrients from living plant cells with active metabolism, prefer juvenile plant tissues. On the contrary necrotrophic pathogens, such as *Botrytis* or *Sclerotinia,* which produce toxins and cell-wall-degrading enzymes to damage the plant cells to get their nutrients, prefer senescent plant tissues. Hemibiotrophic pathogens, such as *Pseudomonas* bacteria or *Phytothora infestans* in their first life cycle, behave as biotrophic and in their second life cycle as necrotrophic pathogens and are, in some respect, between biotrophs and necrotrophs [10,11].

In the next paragraphs, various factors of plant senescence that are supposed to influence the reaction of plants to pathogen infection are listed.

## 2. Factors of Plant Senescence That Can Influence Plant–Pathogen Interactions

**Figure 1:**
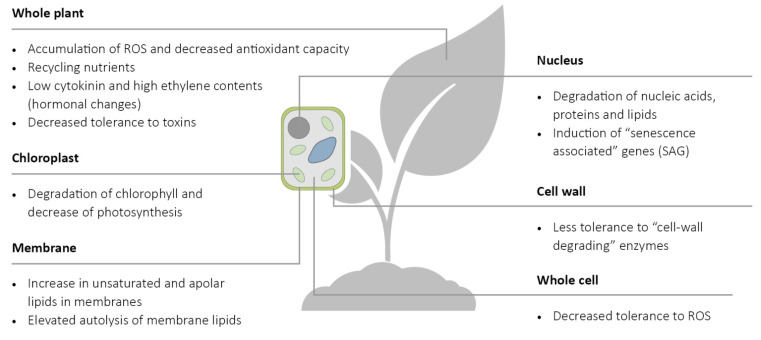
Various factors of plant senescence expressed in whole plant or cell organelles are listed that generally improve plant tolerance to biotrophic and susceptibility to necrotrophic pathogens. Degradation of chlorophyll and macromolecules, accumulation of ROS, and recycling nutrients directly improve plant tolerance to biotrophic pathogens, while decreased tolerance to toxins, to cell-wall-degrading enzymes, to ROS, and to autolysis of membrane lipids directly improves susceptibility to necrotrophic pathogens.

Senescence is a tightly controlled genetic process at the organismal, cellular, and molecular levels. In addition, leaf senescence is an essential process and is a form of programmed cell death [12]. Without to be complete, characteristic features of the senescence process of plants are listed in Figure 1, which can have influence on their resistance to diseases and abiotic stresses.

*Degradation of chlorophylls and decrease of photosynthesis* is unfavourable for biotrophic pathogens since they need active photosynthesis, namely sugars, for their development [5,10,11]. In our early work, we found that not only keeping the leaves in dark but treatment with photosynthesis inhibitors DCMU or CMU retarded development of wheat stem rust; however, adding sugars to the inhibitor solutions reversed the inhibition of rust sporulation [13].

**Figure 2:**
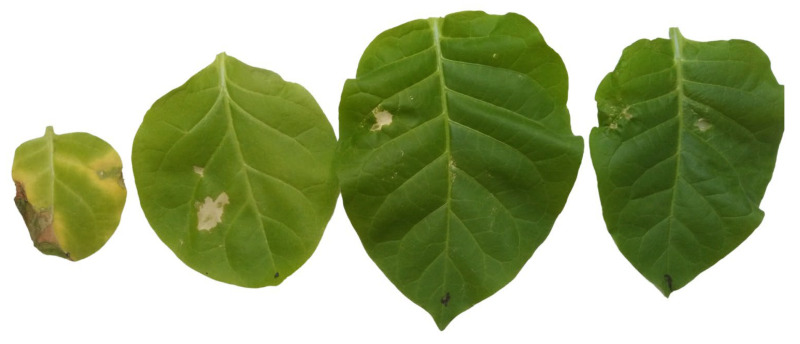
Damage caused by 30% (*v*/*v*) H_2_O_2_ four days after injection into the second, fourth, sixth, and eighth leaves (from the left to the right) of a two-month-old cv. Samsun tobacco plant.

There is a general reaction of plants to abiotic and biotic stresses; a *rapid accumulation of reactive oxygen species (ROS)* [14,15,16] can damage practically all cell constituents, but they can also serve as defence signals [17,18]. The complex role of ROS and the antioxidant systems in disease development and immunity has been discussed several times [19,20,21]. During aging, the antioxidant capacity of plant tissues is generally decreasing [22]. Figure 2 shows that the largest damage is caused by the reactive oxygen H_2_O_2_ on the oldest leaf and the smallest damage on the youngest leaf of the tobacco plant, which indicates the lowest and highest antioxidant capacity, respectively.

Obviously, cell damage caused by ROS is favourable for necrotrophic pathogens, while biotrophs prefer plant tissues with high antioxidant capacity to protect cells from the oxidative damage. Thus, the decreased antioxidant activity of senescent plant tissues is another factor that is favourable for necrotrophs but not for biotrophs [4,21]. To the contrary, the biotrophic barley powdery mildew induced the highest antioxidant activity in the compatible interaction with susceptible barley and not in the incompatible interactions with near-isogenic barley lines carrying various resistance genes, partly to defend itself but also to save the attacked tissue to remain alive and support the biotrophic pathogen [23].

Senescence is an essential physiological process that is accompanied by the *remobilization of nutrients* from senescent leaves to the young organs of the plant [24]. The nutrients derive from the degradation of macromolecules, such as nucleic acids, proteins, and lipids. Since biotrophic pathogens need the active function of these macromolecules, while necrotrophs prefer the above degraded nutrients, obviously, senescence is favourable for necrotrophic pathogens in this respect as well. Moreover, as will be discussed later, biotrophic pathogens redirect the flow of nutrients to the infected part of the plant and the leaves for their own development.

There are several *senescence-associated genes (SAG)* in plants, and many of them are involved in plant immunity [7]. In the past decades, large numbers of senescence-associated genes have been identified [25]. These genes encode various proteins, including RNases, proteases, lipases, transporters, transcription factors, proteins related to translation, antioxidant enzymes, and even pathogenesis-related (PR) proteins [26,27]. Infection of plants by viral, bacterial, or fungal pathogens induces genes that are highly expressed during senescence [28]. It is suggested that the link between defence response and senescence could involve programmed cell death [29]. Among senescence-associated genes, there are many transcription factors (TFs), especially in the WRKY and NAC families, as they have been reported to regulate leaf senescence and plant immunity [7]. It is noteworthy that in addition to the “classical” SAGs, other genes can have strong effect on senescence processes. We found that plant nucleosome assembly protein-related proteins (NRPs) encoding genes, when overexpressed, slowed down development and senescence of Arabidopsis plants, while knockout mutants showed accelerated flowering and leaf senescence. Accordingly, the biotrophic powdery mildew pathogen *Golovinomyces orontii* infection caused the most severe symptoms on the juvenile NRP-overexpressing plants, and the knockout mutants had the least infection density, while infection with the necrotrophic pathogen *Sclerotinia sclerotiorum* induced the most severe symptoms on the knockout mutants, and the over-expresser plants tolerated most of the necrotic symptoms of the *Sclerotinia* infection [30]. 

*Phytohormones* (plant growth regulators) influence practically all aspects of the physiological processes of plants, including senescence and juvenility of plant organs and tissues. We can divide them into two groups: senescence inhibiting and promoting (stress) hormones. To the first group belongs the first of all cytokinins and, in some respects, auxins and gibberellins. The other hormones, in addition to promoting senescence, suppress various stresses and/or are induced by biotic and abiotic stresses. In this group, the two classical hormones, i.e., ethylene and abscisic acid, and the new hormones, i.e., salicylic acid, jasmonate, brassinosteroids, and very recently strigolactones, can be found. During senescence, the contents of cytokinins, gibberellins, and to some extent auxins decrease, while amounts of ethylene, abscisic acid, jasmonates, and salicylic acid increase [31] Furthermore, treating plants with various hormones to alter senescence is a common practice [32].

*Elevated cytokinin and auxin* content have a pivotal role in maintaining juvenility and the active metabolism of plant tissue infected by biotrophs, such as rust or powdery mildew (green island syndrome), and in directing nutrient transport to the infected plant parts [33]. Another definitive but also unique role of the augmented level of cytokinins and auxins is the induction of tumours (crown gall disease) by *Rhizobium radiobacter* (*Agrobacterium tumefaciens*) to form an “opine producing factory” on many economically important crops, which is necessary for this bacterium [34]. 

The relationship between gibberellins and senescence is a little more complex. It is suggested that GAs positively and their signalling components DELLA proteins negatively regulate dark-induced senescence and chlorophyll degradation. Many studies have evidenced that GA modulates plant disease resistance by inducing the degradation of DELLAs, a class of nuclear growth-repressing proteins that act as central suppressors of GA signalling. Negative interaction between GA and DELLA has resulted in resistance response to biotrophic fungus and susceptibility to necrotrophic ones under high GA levels [35,36].

Ethylene, although the simplest plant hormone, has very widespread effects on the physiology of plant, including induction of senescence. In addition, ethylene is an important player in the signal transduction pathways of defence to necrotrophic and hemibiotrophic pathogens together with jasmonic acid [37,38,39,40], which is distinct from the salicylic acid (SA) pathway [41,42,43,44] or even antagonistic to it [45]. SA has a central role in systemic acquired resistance [46,47,48] but is also involved in regulating gene expression during leaf senescence [49]. Similarly, jasmonic acid (JA) was also suggested to be involved in leaf senescence [50]. Furthermore, it was reported that strigolactone regulates leaf senescence in concert with ethylene [51]. Brassinosteroids, in some respect, promote plant senescence [52,53], and additionally, brassinosteroids have strong effect on plant reactions to abiotic and biotic stresses [54,55].

*The fluidity of the plant cell membranes* are decreasing during senescence of plant tissues [56,57,58]. As regards membrane stability, the more rigid membranes with lower polar lipid and unsaturated fatty acid contents of older plant cells are much less tolerant to stresses than the more fluid membranes of younger plant cells, indicating the importance of the physiological state of cell membranes in stress tolerance, which largely depends on its lipid composition. The ratio of polar lipids such as phospholipids and galactolipids to apolar lipids such as sterols and the ratio of the unsaturated to saturated fatty acids in plant cell membranes are gradually decreasing during the aging of plant cells in parallel with their abiotic and biotic stress tolerance [59,60,61]. 

*Phytotoxins* are compounds produced by phytopathogen microorganisms that are toxic in low amount to plants. Thus, neither phytoncides, which are antimicrobial compounds derived from plants, nor mycotoxins, which are derived from plant pathogen microorganisms but are toxic to animals and humans, belong to phytotoxins. It is important to emphasize that phytotoxins are produced only by necrotrophic and hemibiotrophic pathogens since biotrophs do not “want” to kill the plant cell or tissue because they need the living cells. Phytotoxins can be divided into two groups: host-specific (selective) and nonspecific (nonselective) toxins. Host-specific toxins are toxic only to plants that are susceptible to the specific toxin-producing pathogen. Nonspecific toxins can be toxic to any plant without host specificity. Specific toxins can substitute the pathogen; therefore, they are considered as pathogenicity factors, while nonspecific toxins are considered as virulence factors. It was found already a long time ago that senescent plant tissues are more sensitive to both the *host-specific T-toxin* and to the *nonspecific fusaric acid toxin* than to juvenile plant tissues [62].

*Cell-wall-degrading enzymes* (CWDEs) degrade plant cell wall to help pathogens to obtain their nutrients from the cells [63,64]. According to the major cell-wall constituents, they can be divided into pectinases and cellulases (and hemicellulases). They are produced almost exclusively by necrotrophic and hemibiotrophic pathogens and considered to be as important virulence factors of *Erwinia* bacteria, causing soft rot diseases. However, rust infection was also reported to cause augmented cellulase activity as well [65,66]. A long time ago, we could demonstrate that older tobacco leaf tissues are more sensitive not only to phytotoxins but also to cell-wall-degrading pectinase and cellulase enzymes as well as to autolysis of membrane lipids than to younger leaf tissues. Consequently, all the above-mentioned factors of senescent tissues also contribute to their decreased resistance to necrotrophic pathogens [3]

## 3. Effect of Pathogen Infection on the Physiological State of Plants

Although in the past decades, significant progress has been made in the understanding the molecular *mechanisms of plant disease resistance*, still there are many open questions [67,68,69]. It is generally accepted that pattern-triggered immunity (PTI), when a receptor like kinase recognizes a pathogen/microbe-associated molecular pattern (PAMP/MAMP) of the pathogen, or effector-triggered immunity (ETI), when a product of the plant resistance gene directly or indirectly recognizes a specific effector produced by the pathogen, are the major processes to stop the pathogen invasion. The question arises: how can we manipulate the physiological state of plants in order to improve their resistance/tolerance to biotic stresses? However, as we explained in the introduction, changes in the physiological state of plant can influence mainly the expression of disease symptoms, and a fully susceptible plant does not become fully resistant. In spite of this, improvement of the quantitative resistance or tolerance of plants can be very useful in the agricultural practice. 

It has been known for a long time that almost all types of diseases of plants are accompanied by changes of hormone contents and hormonal balance. However, it should be emphasised that in plants, a hormonal crosstalk exists with antagonistic and synergistic effects, which strongly influence their action on disease resistance [41,70,71,72]. Consequently, if someone changes the content of any hormones in the plant, it will influence the action of many other hormones as well. Therefore, it is not enough to determine the amount of a single plant hormone; it is necessary to detect the amount of as many hormones as possible at the same time. As a consequence of the fast development of analytical techniques, recently, it is possible to determine the amount of 15–18 plant hormones and their derivatives from the same plant extracts [73]. Accordingly, we found that the *Obuda pepper virus* (ObPV)-induced hypersensitive response markedly increased not only the levels of salicylic acid (73-fold) and jasmonic acid (8-fold) but also those of abscisic acid, indole-3-acetic acid, indole-3-butyric acid, cis-zeatin, cis-zeatin-9-riboside, and trans-zeatin-9-riboside in the inoculated pepper leaves 3 days post inoculation. On the other hand, the systemic *Pepper mild mottle virus* (PMMoV) infection increased only the contents of gibberellic acid and SA. Hormone contents did not change significantly after ObPV or PMMoV infection in non-infected upper leaves 20 days post inoculation. Concentrations of some brassinosteroids (BRs) and progesterone also increased both in ObPV- and PMMoV-inoculated leaves [74]. Furthermore, when the highly resistant Delisa barley was inoculated with barley powdery mildew, no visible symptoms were found, and only slight changes of hormone contents were detected [75]. Kasote et al. found that jasmonic acid–isoleucine (JA-Ile) and methyl jasmonate (MeJA) were selectively accumulated in fusarium wilt-susceptible and -resistant watermelon plants upon infection [76].

Generally, as it is indicated above, large changes in hormone contents can be found in plants with strong disease symptoms. Phytoplasma diseases are good examples. Several studies have directly or, in many cases, indirectly investigated plant hormone systems in phytoplasma-infected plants. These studies have provided accumulating evidence that phytoplasmas extensively affect plant hormone pathways [77]. In addition, in the literature increasing data indicate that hormones control components of the small RNA system, which regulates many processes (including the siRNA antiviral machinery and the microRNA system) at the transcriptional or post-transcriptional level [78]. Thus, pathogen infections can strongly influence the hormonal network of plants in order to support their own development [79]. 

## 4. Manipulation of the Physiological State of Plants in Order to Improve Biotic and Abiotic Stress Tolerance

**Figure 3:**
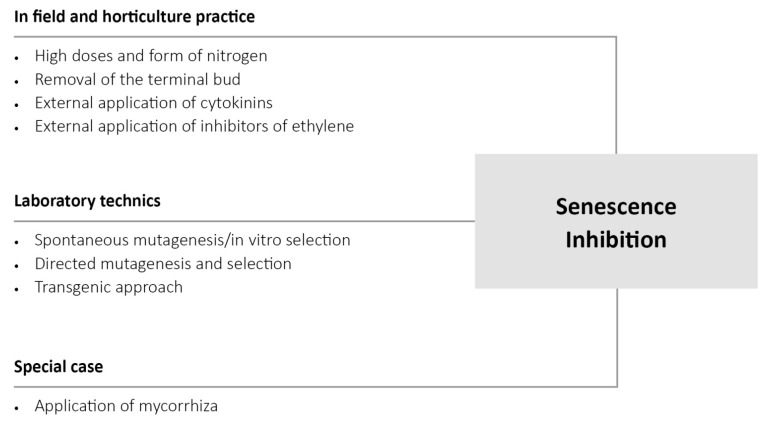
List of various methods for inhibition of senescence to obtain juvenile plants that increase not only yield but also resistance to necrotrophic pathogens. While some methods have been used in the field and in horticulture, other methods need laboratory techniques, and involve direct changes in the plant genome. Application of mycorrhiza is a special case, when in addition to inhibition of senescence additional factors improve resistance to pathogens.

Any change in the physiological state (senescence/juvenility) and hormone balance of plants could influence their tolerance to pathogens. The senescence process of plants can be inhibited by several ways, such as decapitation of plants (removal of terminal bud) or high doses of nitrogen and external application of cytokinins or of inhibitors of ethylene action. In addition, senescence of plants can be altered by mutation and by transgenic approach (Figure 3). Even inoculation with mycorrhiza can improve stress tolerance of plants.

It has been known for a long time that *doses and form of nitrogen nutrition* have strong effect on plant diseases [80,81,82,83]. High doses of nitrogen induce elevated cytokinin activity [84] and increase tolerance to necrotrophic pathogens, such as fusarium wilt [85,86]. On the other hand, elevation of nitrogen doses increases susceptibility to the biotrophic pathogens rusts and powdery mildews [87,88,89]. 

*Removal of the terminal bud,* which disrupts the apical dominance, is an often-used method in horticultural practice to inhibit senescence of plants. We found that this type of decapitation of tobacco plants reduced the number and size of necrotic lesions caused by TMV infection (increased resistance), and in parallel, juvenility was induced that could be detected in the membrane lipid composition as well [60].

There are many examples of the disease reduction by *cytokinin treatment* of plants [2,90,91,92,93] and even resistance to insects can be improved by cytokinin treatment [94,95]. However, changes in a hormone content affect not only the other hormones but many physiological processes, signal transduction pathways, and gene expressions that can act not only synergistically but antagonistically as well. On the other hand, resistance of a plant to a pathogen should be separated from tolerance to disease symptoms. Accordingly, necrotic symptoms of TMV could be suppressed by kinetin treatment of tobacco leaves, but virus multiplication around the lesions was promoted [90]. 

Furthermore, inhibition of senescence by *external application of inhibitors of ethylene action* can also elevate plant tolerance to diseases [96]. Externally applied aminoethoxy-vinylglycine (AVG), an inhibitor of ethylene biosynthesis, not only effectively inhibited ethylene formation but also lesion development by TMV infection. Interestingly, in leaves with 100% damage (frost injury), the inhibitory effect of AVG was much weaker and similar to the effect of propyl gallate (PG), a free radical scavenger [97]. Preharvest application of ethylene inhibitors can be used for modulation of post-harvest fruit and vegetable quality [98]. Similarly, ethylene scavenger techniques are often used in delaying the ripening of fruits and vegetables [99,100]. 

*In vitro selected* paraquat (reactive oxygen producing herbicide) -tolerant tobacco with delayed senescence showed tolerance not only to *Botytis cinerea*, *Alternaria alternata*, and *Tobacco necrosis virus* (TNV) infections but also to fusaric acid toxin and freezing and heat stress [4]. The paraquat-tolerant plants had elevated cytokinin content [101] and augmented antioxidant activity [102]. Even “natural transgenic” tomato plants, which were transformed by the wild type of agrobacterium T-DNA, showed elevated antioxidant enzyme activities, membrane lipid composition of juvenile plants, in addition to disease resistance [61]. The higher cytokinin content in tomato plants that were regenerated from crown galls was due to the activation of the isopentenyl transferase (ipt) gene from T-DNA and was determined by indirect ELISA test [101]. Several mutant plants with inhibited senescence have elevated tolerance to biotic and abiotic stresses. Hirsch et al. [103] showed that *ein2-1*, an *Arabidopsis* ethylene-insensitive *mutant*, expressed delayed symptom development in response to bacterial wilt caused by *Ralstonia solanacearum.* Furthermore, in accordance with the delayed leaf senescence and higher antioxidant activity of *Arabidopsis*, *ore1*, *ore3*, and *ore9* mutants proved to be tolerant to oxidative stress [104]. In addition, it was reported that mutation of the Arabidopsis NAC016 transcription factor delays leaf senescence [105].

**Figure 4:**
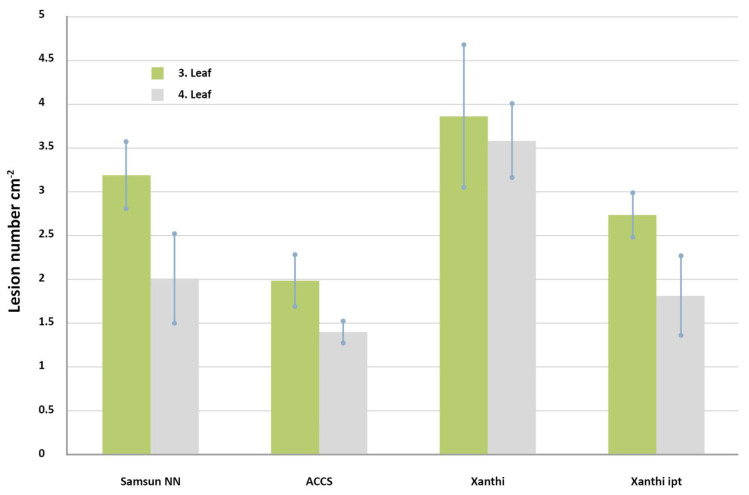
Effect of *Tobacco mosaic virus* (TMV) infection (lesion cm^−2^) on third and fourth leaves of control (Samsun NN and Xanthi nc) and limited-ethylene-producing (ACCS) [107] and cytokinin-overproducing (Xanthi ipt) [109] transgenic tobacco plants. Note that virus lesion number can be significantly decreased similarly by either decreasing ethylene production or increasing cytokinin production in tobacco plants. It is also remarkable that third older leaves always showed more TMV lesions than the fourth younger ones.

In addition to the external application of hormones, in vitro selection, or mutagenesis, a *transgenic approach* has been used to inhibit plant senescence. It is reported that the never-ripe tomato mutant impaired in ethylene perception exhibited a significant reduction in disease symptoms in comparison to the wild type after inoculations with virulent bacterial (*Xanthomonas campestris* pv. *vesicatoria* and *Pseudomonas syringae* pv. *tomato*) and fungal (*Fusarium oxysporum* f. sp. *lycopersici*) pathogens. Bacterial spot disease symptoms were also reduced in tomato genotypes impaired in ethylene synthesis and perception [106]. However, it has to be emphasized that the effect of ethylene (and other hormones) on plant disease resistance is also controversial [37,107]. We found that the HR symptoms were decreased, but the multiplication of *Pseudomonas* bacteria was elevated in cytokinin-overproducing tobacco as compared to the control non-transformed ones [108]. Furthermore, one can produce similar improvement of stress resistance if senescence is suppressed by overproducing cytokinin activity or inhibition of ethylene action, as it is illustrated by the elevated tolerance of both of these tobaccos to TMV infection. It is also noteworthy that the younger fourth leaves showed fewer lesions than older third leaves (Figure 4).

Another transgenic approach is directly improving the antioxidant capacity of plants. Overexpression of ROS scavenging enzymes such as isoforms of SOD (Mn-SOD, Cu/Zn-SOD, Fe-SOD), CAT, APX, GR, DHAR, GST, and GPX resulted in abiotic stress tolerance in various crop plants due to efficient ROS scavenging capacity. Pyramiding of ROS scavenging enzymes may also be used to obtain abiotic stress-tolerance plants [110,111,112]. 

A special case is the group of lesion mimic mutant (LMM) plants, which show early senescence [113,114]. LMM plants develop HR-like necrotic lesions without pathogen attack, and this type of programmed cell death (PCD) often induces expression of pathogenesis-related (PR) protein genes [115]. However, the elevated resistance of LLM plants to facultative pathogens can be variable [116]

An additional way of inhibition of senescence and of induction of resistance to pathogens via improving the physiological state of plants is *inoculation with mycorrhiza* [117,118]. The topic would need a separate review; therefore, we only mention some works dealing with the endophytic fungus *Piriformospora indica*. This basidiomycete promotes plant growth, increases yield, and induces not only resistance to various pathogens but tolerance to salt stress as well. The resistance/tolerance of *P. indica*-inoculated plants is associated with augmented antioxidant activities [119,120,121]. 

## 5. Concluding Remarks

The physiological state, i.e., senescence or juvenility, of plants has a significant effect on their reactions to pathogen attacks, expressed as changes in the severity of disease symptoms. Generally, necrotrophic pathogens cause more severe symptoms on senescent than on juvenile plant tissues, while biotrophs prefer juvenile plants since several factors of senescence have an opposite effect on the susceptibility of plants to necrotrophic and biotrophic pathogens.

The plant hormonal network, with antagonistic and synergistic effects, strongly influences the physiological state and disease tolerance of plants. The pathogen infection, depending on the lifestyle of the pathogen, always changes hormone contents and balance in plants to favour its own development.

There are several possibilities to manipulate the physiological state of plants in order to improve their biotic and abiotic stress tolerance, such as removal of terminal bud or high doses of nitrogen and external application of cytokinins or of inhibitors of ethylene action as well as by mutation, in vitro selection, by transgenic approach, or even by applying mycorrhiza.
